# Supplementary material for: Severe acute malnutrition among children under the age of 5 years
Source: PLoS One. 2024 Aug 26;19(8):e0309122. doi: 10.1371/journal.pone.0309122 (PMC11346641; doi:10.1371/journal.pone.0309122)
Supplement: Attachment — Submitted filename: RESPONSE TO REVIEWERS 002.pdf [file pone.0309122.s006.pdf]

Email: smasenga@mu.ac.zm
website: www.mu.ac.zm

Contacts: +260966674774
+260977674774

Mulungushi University
School of Medicine and Health Sciences
HAND Research Group
P.O Box 60009, Livingstone Campus.

2nd July, 2024

To the reviewers and Editor,

**Ref: RESPONSES TO REVIEWER'S COMMENTS**

**We would like to thank the reviewers for taking the time to make suggestions that have improved our manuscript. We have now made revisions to the minor comments in the manuscript and incorporated all suggestions. We now hope the current manuscript is acceptable for publication. Below are the point-by-point responses to all comments and suggestions.**

**Reviewer #1: General comment**
Thank you for considering the suggestion on the inclusion of the participants' flow chart. According to the strobe guidance flow charts describing the participants should be included in the results sections

Response: We have now moved the flow chart to the results section. Thank you for this suggestion

Table 1: Include the means of the haematological parameters comparing those with SAM vs No SAM

Response: We have included them. Thank you.

Could the authors review the confidence intervals for the adjusted analysis for the platelet count in table 2?

Response: The confidence interval are 1.000 (1.000, 1.001) so we just rounded them off to 2 decimal places to maintain consistence with other values in the table. For clarity, we have included this in the footnote. Thank you.

Reviewer #2: The overall quality of the revised manuscript improved a lot. The authors should be congratulated for that. However, I have minor concerns about the following three definitions that need to be revised before the acceptance of this manuscript.
1. The authors mentioned that clinical diagnosis of TB was made when bacteriological criteria were not met but the authors did not mention the clinical criteria used for clinical TB diagnosis. These criteria should be precisely written.

Email: smasenga@mu.ac.zm
website: www.mu.ac.zm

Contacts: +260966674774
+260977674774

Mulungushi University
School of Medicine and Health Sciences
HAND Research Group
P.O Box 60009, Livingstone Campus.

Response: Thank you for your kind remarks. We have now written the clinical diagnosis. The criterion included a medical history, TB contact, physical examination, chest x-ray and a positive tuberculin skin test). Thank you!

2. The authors defined acute watery diarrhea as ‘frequent loose or watery stools lasting less than 2 weeks’ which is not precise as this should be defined as ‘more than three loose or watery stools per day lasting less than 2 weeks (WHO Pocket Book 2013, 2nd edition, page 125)’

Response: Thank you for the precise definition. We have now included it.

3. The authors defined pneumonia as ‘a lung infection caused by bacteria or viruses, diagnosed using either a chest radiograph or clinically through vital sign measurement’, this is not a precise definition at all. The authors should use the better reference (PMID: 15976876) for chest radiograph defining pneumonia and must need to mention the clinical features used for diagnosing pneumonia/severe pneumonia. The authors may take the help from WHO Pocket Book 2013, 2nd edition, page 80).

Response: Thank you for suggesting the precise definitions. We have now included. Much appreciated.

We have revised the manuscript and addressed all concerns raised by the reviewers. We want to thank you all again for the tremendous work and time that you committed in editing our work. Our manuscript is much improved, and we are very grateful.

Yours sincerely,

**Sepiso K. Masenga**, BSc., MSc., PhD., FGHF., PGDip

Associate Professor of Human Pathology,
Chair/Director of Hypertension, HIV/AIDS, Nutrition, Diabetes and Dyslipidemia (HAND) Research Group
Coordinator Postgraduate programs
Head of Physiological Sciences Department
Academic Editor – Frontiers in Cardiovascular Medicine
Editorial board member - PLOS ONE
Editorial board member – BMC infectious diseases
