# Supplementary material for: Severe acute malnutrition among children under the age of 5 years
Source: PLoS One. 2024 Aug 26;19(8):e0309122. doi: 10.1371/journal.pone.0309122 (PMC11346641; doi:10.1371/journal.pone.0309122)
Supplement: Attachment — Submitted filename: PlosOne SAM ZM review comments.docx [file pone.0309122.s003.docx]

**General comment**

This paper describes the prevalence and identifies factors associated with severe acute malnutrition (SAM) among children <15 years in a regional hospital in Zambia. This is indeed a topic of public health importance given that severe malnutrition is the underlying cause of several childhood morbidities and contributes to mortality in Africa and other lower-resource settings.

The main problem of this paper is how the definition of SAM is applied in the study. Whereas the WHO definition of SAM applies to children between 6 months <5 years with specific criteria (presence of either WHZ <-3 standard deviation or MUAC<115mm or bilateral pitting oedema) the authors have applied only one of these parameters to define SAM, and have broadly used the definition to include children =>5 years with BMI for age <-3 SD who although technically are severely undernourished but they do not meet the definition of SAM.

I suggest the authors apply the definition as per WHO definition to children 6 months < 5 years and only include children in this age category in their analysis or use the term that can broadly encompass all forms of malnutrition or undernutrition if they wish to understand malnutrition more broadly in children <15 years.

Below are specific recommendations for each section:

**Abstract**

Background: Include a statement with the aim or hypothesis of the study.

Methods: Provide the definition of SAM and state inclusion criteria in this section

Results: Provide a summary of how many records were screened, and found eligible and clearly state the number of children the analysis is based on.

**Introduction**

Paragraph 3, Line 1: Review figures for the prevalence of undernutrition in Zambia which are much lower than those provided in the Zambia Demographic and Health Survey (<https://www.zamstats.gov.zm/portfolio/zambia-demographic-and-health-survey-zdhs/>) which provides a more countrywide representative figure than the reference used.

**Methods**

#Study design and setting:

A brief description of the hospital and the type of population served by the facility would help understand the settings in which this study was conducted.

#Eligibility/sampling:

Explicitly state what the study inclusion and exclusion criteria used. Were the participants selected hospitalised or recruited from ambulatory settings as well?

#Variables:

As stated in the general comments, the WHO criteria for SAM is: WHZ<-3 or MUAC<115 or the presence of bilateral pitting oedema in children 6mo <5years. Technically BMI for age <-3 in children above 5 years is described as “severe thinness” (<https://www.who.int/tools/growth-reference-data-for-5to19-years/indicators/bmi-for-age> )and is not interchangeable with SAM although it does provide a measure of severe undernutrition in this age group.

Were the classifications of SAM/malnutrition based on clinician diagnosis or the parameter (WHZ, BMI for age) calculated by the investigators using the clinical data? How were the data on the other parameters for SAM (MUAC or pitting oedema) dealt with?

\# Sample size calculation:

- What was the rationale for inclusion of 10% non-response rate in a study that was primarily a retrospective review of patient records?

#On data collection:

- The procedures for data collection should be sufficiently described. How was the review of medical records structured? Was the SAM information extracted from the most recent medical visit or did it include any previous hospital visit? If there were multiple visits which instance was used?
- How was the screening of SAM structure in this institution? Do all children attending the hospital get a weight, height, and MUAC measurement?
- Did this review include hospital records over the stated study period only? If yes, how many <15 years were seen over this period in the hospital?

**Results:**

#Baseline characteristics

- Provide a summary of how many participant records were screened, found eligible, included, and analyzed. A flow chart could provide reason for exclusions of medical records.
- How many medical records were included in this analysis? Every 2nd record from a sample of 3000 implies there were 1,500 screened. How were the 695 finally selected for inclusion?
- Provide summary statements of the measures of malnutrition (WHZ, BMI) in the text or Table 1 to help understand the general nutritional status of the study cohort.
- The HIV prevalence among participants was approximately 9.2% (n=62). What was the denominator used here? 62/695 = 8.9%
- How many cases of tuberculosis were in this cohort? It’s not explicitly stated the number of tuberculosis cases in this cohort but only the proportion with pulmonary disease is provided in line 5 of the results.
- Both tables included are not referenced in the text in the results section to allow the reader to refer to the appropriate table.
- Table 1: Could you include how many TB cases were confirmed(bacteriologically) within the table or in the footnote?

**Discussion:**

- In paragraph 2, on the association between HIV and malnutrition, the authors reference adult studies only and do not refer to any relevant pediatric studies.
- In paragraph 6, apart from the retrospective nature of the study, where there any other limitations? How about the methods used to identify children with SAM? Some discussions on the limitations of the methods used to identify children with SAM should be included in this section.
- What is the implication of the findings for the care of children with malnutrition in this region or setting?
