# Supplementary material for: Severe acute malnutrition among children under the age of 5 years
Source: PLoS One. 2024 Aug 26;19(8):e0309122. doi: 10.1371/journal.pone.0309122 (PMC11346641; doi:10.1371/journal.pone.0309122)
Supplement: Attachment — Submitted filename: Review reports_PONE-D-24-15995.doc [file pone.0309122.s004.doc]

Date: 01/06/2024

To: Professor Stephen Michael Graham

The Academic Editor, The PLOS ONE

From: Mohammod Jobayer Chisti

Senior Scientist & Head, Clinical Research, Hospitals, icddr,b, Dhaka, Bangladesh

Subject: Peer review on the manuscript entitled “Prevalence and Factors associated with Severe Acute Malnutrition among children under the age of 15 years”.

Dear Professor Stephen Michael Graham,

Thanks for kindly providing me the opportunity to review the above-mentioned mentioned manuscript. I have provided my review comments below.

**General comments**:

The authors have chosen an important topic with high public importance and written the manuscript evaluating the prevalence and factors associated with SAM among children under the age of 15 years in an African country. The authors performed this study among under-15 children instead of under-5 children, although no rationality or data in favor of conducting this study in under-15 children are shown. The rationality should be elaboratively discussed in the background and briefly in abstract and need to have reflection all through the manuscript.

**Specific comments**:

**Abstract**

1. Please provide brief justification of conducting this study in under-15 children in the background

2. Please define SAM briefly in the methods

3. Usually abstract stands alone and thus before providing 16.7% prevalence in the results of abstract, the authors should provide the exact denominator and numerator here.

**Background**

4. Burden and fatality of SAM and related references used here are old and there are a number of recent data on these especially after 2022 and the authors should provide the most recent references all through the background.

5. The authors are requested to provide strong justification of conducting this study in under-15 children and need to have argument on how under-5 SAM aligns/differs to/from each other.

6. The background needs to have more connectivity with aims/objectives in the last paragraph.

**Methods**

*Sample size*

7. The evidence of 5% prevalence of SAM is used in calculating sample size in the Gambia, however, the prevalence was as high as 11% (<https://www.lshtm.ac.uk/research/units/mrc-gambia/news/321426/mrc-nutrition-rehabilitation-centre-caring-children-severe-acute-malnutrition>). The authors need to justify the selecting the lower prevalence for calculating the sample size.

*Variables*

8. There should have a brief description (a paragraph) on the procedure of the anthropometric measurement (weight, length/height, BMI) in children

9. Please correct acute diarrheal diarrhea as acute watery diarrhea with its definition

10. Please also define anemia, pneumonia, sickle cell disease, tuberculosis (bacteriological/clinical)

*Data collection and data analysis*

11. As data is a plural noun, please revise the grammar accordingly all through the text.

**Results**

Basic characteristic of the study participants

12. Definition of MAM should be provided in the methods before its use in the results.

13. Elaboration of SAM has already used earlier in the text, thus, this is redundant here.

14. All statistical values shown in the text of the results (Basic characteristic of the study participants and Univariable and multivariable analysis of factors associated severe acute malnutrition) are also available in the relevant tables. Please refer the tables for the statistical values rather duplicating these values in the text.

**Discussion**

15. Again, the elaboration of SAM has already used earlier in the text, thus, this is also redundant here.

16. The discussion section is less organized especially this section need to focus on most important observation with more explicit evidences.

17. The authors need to provide argument with evidences on how under-5 SAM aligns/differs to/from each other.

18. The observation of the association of an increase in neutrophil count with decreased odds of SAM should be discussed with more insights especially with the relation poor inflammatory responses in SAM.

18. Conclusion should be revised (deducting severe acute malnutrition or SAM) making a link with implication of the study.
